# Differentiable Neural Substrates for Learned and Described Value and Risk

**DOI:** 10.1016/j.cub.2010.08.048

**Published:** 2010-10-26

**Authors:** Thomas H.B. FitzGerald, Ben Seymour, Dominik R. Bach, Raymond J. Dolan

**Affiliations:** 1 Wellcome Trust Centre for Neuroimaging, 12 Queen Square, London WC1N 3BG, UK; 2 Department of Clinical Neurosciences, Institute of Psychiatry, Camberwell, London SE5 8AF, UK; 3 Economic and Social Research Council Centre for Economic Learning and Social Evolution, Gower Street, London WC1E 6BT, UK

## Abstract

Studies of human decision making emerge from two dominant traditions: learning theorists [1–3] study choices in which options are evaluated on the basis of experience, whereas behavioral economists and financial decision theorists study choices in which the key decision variables are explicitly stated. Growing behavioral evidence suggests that valuation based on these different classes of information involves separable mechanisms [4–8], but the relevant neuronal substrates are unknown. This is important for understanding the all-too-common situation in which choices must be made between alternatives that involve one or another kind of information. We studied behavior and brain activity while subjects made decisions between risky financial options, in which the associated utilities were either learned or explicitly described. We show a characteristic effect in subjects' behavior when comparing information acquired from experience with that acquired from description, suggesting that these kinds of information are treated differently. This behavioral effect was reflected neurally, and we show differential sensitivity to learned and described value and risk in brain regions commonly associated with reward processing. Our data indicate that, during decision making under risk, both behavior and the neural encoding of key decision variables are strongly influenced by the manner in which value information is presented.

## Highlights

► Learned and explicitly described value and risk have different effects on behavior ► Learned and described value and risk have separable neural correlates ► Learned and described value are traded off in several brain regions ► Activity in the orbitofrontal cortex predicts bias toward learned options

## Results and Discussion

### Experimental Paradigm

**Figure 1:**
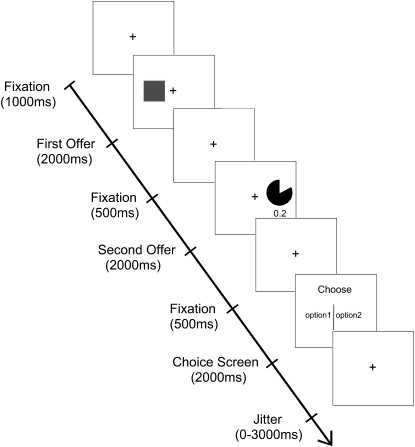
Illustration of a Single Trial of the Task Paradigm Subjects fixate for 1000 ms. They are then presented with the first offer (which can be either a described-value cue or a learned-value cue, fully counterbalanced and in pseudorandomized order) for 2000 ms, and, after a 500 ms delay, the second offer for 2000 ms. After another 500 ms delay, they are then asked to make their choice within 2000 ms. Successful choices were indicated by the appearance of a circle around the selected option. The intertrial interval was jittered between 0 and 3000 ms. In the example shown, the subject is being asked to decide between the option indicated by the square (the value of which they have previously learned) and a described-value option with a win probability of 0.2. (In this paradigm, outcome magnitudes are held constant, and so probability and value are equivalent.) For the fMRI analysis, events were modeled at both the first offer and choice-screen onset time.

We used an event-related fMRI paradigm in which subjects (n = 17) made choices between three cues whose win probability they had previously learned (p = 0.1, 0.5, 0.9) and cues whose values were described in terms of an explicit win probability (nine cues, p = 0.05, 0.1, 0.2, 0.4, 0.5, 0.6, 0.8, 0.9, 0.95) (Figure 1). Probabilities were described both numerically and with the aid of a pie chart (note that because we only manipulate probability, and not magnitude, probability and value are effectively equivalent in our study). We then applied a logit analysis to subjects' choice patterns to derive estimates of the subjective value of the learned cues in terms of explicit probabilities [9, 10]. We hypothesized that brain activity in regions associated with reward processing, specifically ventromedial prefrontal/medial orbitofrontal cortices (vmPFC/OFC), posterior cingulate cortex (PCC), and ventral striatum (VS), would show differential patterns of activity when subjects processed experienced and described values, respectively [11–15].

### Behavioral Findings

**Figure 2:**
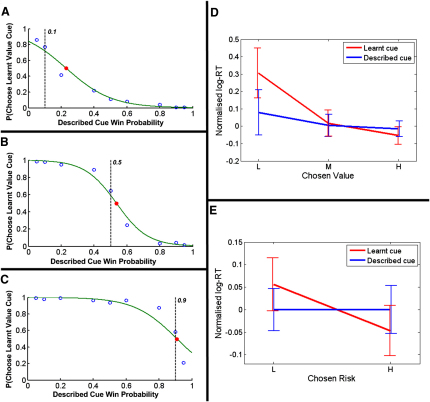
Behavioral Analysis (A–C) Logit analysis of subjects' pooled choice data for the lowest learned-value cue (p = 0.1) (A), the middle learned-value cue (p = 0.5) (B), and the highest learned-value cue (p = 0.9) (C). The probability of subjects choosing the learned-value option, when it was offered against each separate described-value option, was calculated (indicated by open blue circles), and this resulting probability distribution was fitted with a logistic sigmoid (green line). The indifference point (red filled circle) calculated from this was used as an estimate of relative subjective value (in other words, an estimate of each learned-value cue in terms of described probabilities). Indifference points were p = 0.23, 0.54, and 0.91, respectively, suggesting that subjects considerably overvalued the lowest learned-value cue. This can be seen by comparing the actual estimated subjective values (red filled circles) with the normative ones (indicated by dotted vertical lines) and suggests that subjects exhibited a bias toward learned options when considering low value alternatives. (Note that for the imaging analysis described here, individual subjects' choice patterns were analyzed separately.) (D) Normalized log reaction times (RTs) pooled across all subjects from which the effects of experimental session, difference in subjective value, and selected option type have been regressed out (Supplemental Experimental Procedures). These have been binned into low, medium, and high chosen subjective value. Blue lines indicate mean log RTs from trials in which subjects chose the described-value cue, red lines indicate those in which they chose the learned-value cue, and vertical bars indicate 90% confidence intervals. The figure illustrates that RTs were negatively correlated with increasing chosen value only if subjects chose a learned-value cue, showing no equivalent effect of described value. (E) Normalized log RTs pooled across all subjects from which the effects of experimental session, difference in subjective value, selected option type, and chosen value have been regressed out (Supplemental Experimental Procedures). These have been binned into low and high chosen subjective risk. Blue lines indicate mean log RTs from trials in which subjects chose the described-value cue, red lines indicate those in which they chose the learned-value cue, and vertical bars indicate 90% confidence intervals. The figure illustrates that RTs were negatively correlated with increasing chosen risk only if subjects chose a learned-value cue, showing no equivalent significant effect of described risk.

Our behavioral results, evident in both subjective valuation and reaction time (RT) data, were consistent with learned and described values being processed differently during choice. Subjects significantly overvalued low (but not medium or high) learned-probability relative to described-probability cues (p < 0.005 two-tailed t test; Figures 2A–2C; see also Table S1A available online). This suggests that, for low win probabilities, the effect of learned value (LV) on choice was stronger than that of described value (DV), congruent with previous findings about explicit estimation of learned outcome probabilities [16] (Supplemental Data).

Superficially, our behavioral findings seem to contradict evidence suggesting that low described probabilities tend to be overweighted and low learned probabilities underweighted [7]. In fact, we believe there is no such contradiction, because major procedural differences, most notably the focus of previous studies on testing probability weighting within domain, with subjects choosing between pairs of learned-probability options or pairs of described-probability ones, are likely to account for any apparent difference. In our task, subjects were required to compare valuations across domains—in other words, to make a choice between a learned-probability option and a described-probability option. Because all subjects received the same amount of feedback about each learned cue, our data also suggest that behavioral differences in handling learned and described probabilities are unlikely to be due solely to sampling bias [7].

A multiple regression analysis of RT data showed no significant effect of either choice condition (whether subjects chose the learned- or described-value cue) or the subjective value of the chosen option. Importantly, there was a significant RT choice-condition-by-value interaction (p < 0.01), indicating that learned value facilitated behavioral responding, whereas described value did not (Figure 2D; Table S1B). This effect of learned value is entirely consistent with a well-established facilitative effect of appetitive conditioning on reaction times [15, 17].

### Brain Responses to Value

Our use of a sequential presentation paradigm allowed us to examine value-correlated activity at separate times during the trial. Here our primary focus is on value signals present at choice-screen onset (reflecting the value signals present during actual choice), but we also consider neural activity at the presentation of the first offer to the subject (representing initial encoding and evaluation of stimuli; Supplemental Experimental Procedures; Supplemental Data). In addition, cognizant of the fact that neuronal processes involved in valuation might change as a function of time, we tested for temporally decaying value signals at both time points (Supplemental Experimental Procedures; Supplemental Data).

**Figure 3:**
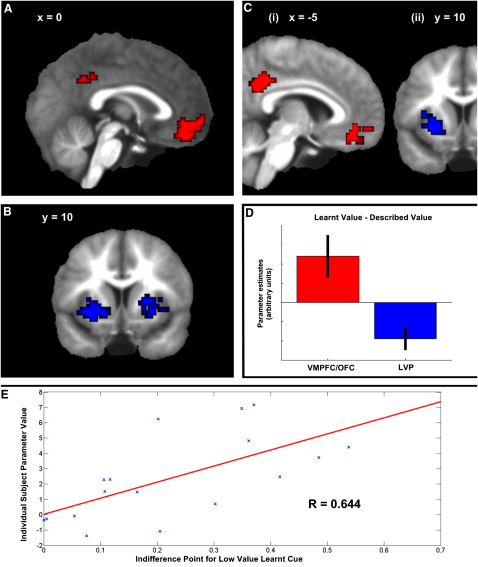
Neural Correlates of Learned and Described Value at Choice Time (A) This shows that learned value correlated with activity in the ventromedial prefrontal/medial orbitofrontal cortices (vmPFC/OFC) and posterior cingulate cortex (PCC). vmPFC/OFC: peak cluster voxel ([−15, 57, −3], z = 4.20), p < 0.002 whole-brain cluster corrected. PCC: peak voxel ([−3, −48, 33], z = 2.62), p < 0.05 cluster corrected for the PCC region of interest (ROI). Image is at x = 0. (B) Described value correlated with activity in the bilateral ventral putamen (VP). Peak voxels: ([−30, 12, −6], z = 4.29) and ([27, 0, 6], z = 3.60). Both p < 0.002 whole-brain cluster corrected. Image is at y = 10. (Ci) A direct comparison between responses to learned and described value (the (LV – DV) contrast) shows that activity in the vmPFC/OFC and PCC was greater for learned than described value. vmPFC/OFC: peak cluster voxel ([−15, 57, −3], z = 3.66), peak voxel within ROI ([−3, 45, −21], z = 2.73), p < 0.03 cluster corrected for the vmPFC/OFC ROI. PCC: peak voxel ([−9, −48, 30], z = 3.22), p < 0.02 whole-brain cluster corrected. This shows that value-sensitive activity in these regions was selective for learned-value options. Image is at x = −5. (Cii) The opposite (DV – LV) contrast shows that activity in left VP was better correlated with described relative to learned value. Peak voxel: ([−27, 9, 0], z = 3.17), p < 0.03 whole-brain cluster corrected. This shows that value-sensitive activity in these regions was selective for described-value options. Image is at y = 10. (D) Mean parameter estimates for activation in the vmPFC/mOFC (red) and left VP (blue) for the (LV – DV) contrast. This illustrates that activity in the vmPFC/OFC was correlated more strongly with the value of learned cues than described ones, whereas the left VP showed the opposite pattern (this presentation is for illustrative purposes only; black bars indicate 90% confidence intervals). (E) Plot of individual subjects' parameter estimates for the (LV – DV) contrast in the vmPFC/OFC (y axis) against their estimated subjective value for the lowest learned-value cue (objective win probability = 0.1; x axis). These show a strong positive correlation (R = 0.644, p < 0.01, permutation test), indicating that subjects that showed a greater degree of sensitivity to learned value relative to described value in the vmPFC/OFC also showed a bias toward selecting learned-value options. (Red line indicates the line of best fit generated by linear regression.)

At choice time, we observed activity correlating with learned value in the vmPFC/OFC (p < 0.002 whole-brain cluster corrected) and PCC (p < 0.05 region of interest [ROI] cluster corrected; Figure 3A; Table S2). By contrast, described value was correlated with activity in bilateral ventral putamen (VP) and cerebellum (all p < 0.002 whole-brain cluster corrected; Figure 3B; Table S2). Critically, a direct contrast showed that these activation patterns differed significantly. The (LV − DV) contrast showed differential activity in vmPFC/OFC (p < 0.03 ROI cluster corrected) and PCC (p < 0.02 whole-brain cluster corrected; Figures 3Ci and 3D; Table S2). Conversely, the opposite (DV − LV) contrast was associated with differential activity in the left VP (p < 0.03 whole-brain cluster corrected) and the thalamus (p < 0.002 whole-brain cluster corrected), with activity also evident in the right VP, albeit not reaching our criterion level of significance (Figures 3Cii and 3D; Table S2). Of note, both LV-correlated activity in the vmPFC/OFC and DV-correlated activity in the VP survived in a check model in which learned and described value regressors were orthogonalized to a simple binary choice parameter. These activation patterns, in regions repeatedly implicated in studies of value (e.g., [11–15]), thus reflect option values rather than just selected option type. We emphasize that our findings do not conflict with an established relationship between activity in VS and reward learning [15, 16, 18, 19]. In our paradigm, learning about reward contingencies was asymptotic: subjects merely retrieved previously learned information. LV- and DV-correlated activity at offer time also differed from one another markedly, although the regions involved were different to those involved at choice time (Supplemental Data).

At both choice and offer time, we found regions where activity significantly correlated with both LV and (LV − DV) on the one hand and both DV and (DV − LV) on the other. This raises the possibility that, rather than separately encoding LV and DV, these regions actually process relative value signals (LV − DV) and (DV − LV). Thus, rather than anatomically dissociated networks processing different kinds of reward information, the activity patterns we observe might reflect differential processing of reward information within a distributed value-sensitive network.

In an exploratory post hoc ROI analysis, we addressed this issue by assessing whether activity in regions showing significant responses to the (LV − DV) contrast showed significant negative responses to DV in addition to positive LV responses. We then performed a similar analysis for the (DV − LV) contrast. Note that because we do not make use of unbiased ROIs, any results should be seen as suggestive rather than conclusive. At choice time, a significant negative correlation with DV was found in the PCC (p = 0.009) and with LV in the VP and thalamus (VP: p = 0.046, thalamus: p = 0.007; Figure S3B). A negative correlation with LV was found in vmPFC/OFC, but this was not significant (vmPFC/OFC: p = 0.291; Figure S3B). These findings provide suggestive evidence that activity in PCC and thalamus is sensitive to both LV and DV, though in distinct ways, together with weaker evidence that the same considerations apply to activity in VP and vmPFC/OFC. Based on these findings, we suggest that our results are best seen as reflecting differential sensitivities to different kinds of reward information within a valuation network [11–15], with the establishment of the precise nature of these differences remaining an issue for future work. We note also evidence of relative value coding in a number of regions at offer time (Supplemental Data).

Additionally, we hypothesized that between-subject variability in responses to learned and described value would predict the degree to which individuals displayed choice behavior biased toward selecting learned-value options. This is precisely what we found (Figure 3E; Supplemental Data). Individual subjects' parameter estimates in the vmPFC/OFC for the (LV − DV) contrast showed a significant positive correlation with the extent to which they overvalued the low-probability learned cue (*R* = 0.644, p = 0.012, permutation test). Post hoc testing showed both a strong positive correlation between overvaluing and LV parameter estimates (*R* = 0.482, p = 0.021, permutation test) and a strong negative correlation between overvaluing and DV parameter estimates (*R* = −0.419, p = 0.040, permutation test). This suggests that subjects who showed greater (though opposite) responses to LV and DV in the vmPFC/OFC showed an increased bias toward selecting learned-value options.

### Risk Processing

**Figure 4:**
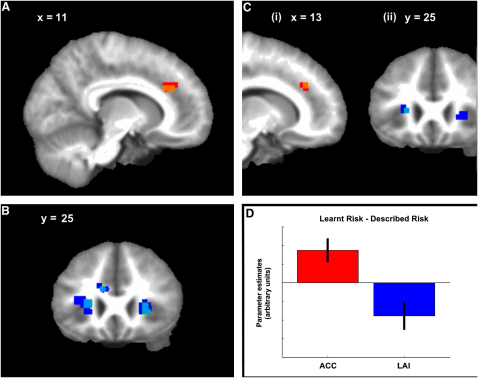
Neural Correlates of Learned and Described Risk at Choice Time (A) The risk of chosen learned options was correlated with activity in the anterior cingulate cortex (ACC). Peak voxel: ([12, 30, 27], z = 3.52), p < 0.05, family-wise error, small-volume corrected (FWE-SVC). Image is at x = 11; red p < 0.005, orange p < 0.001. (B) The risk of chosen described options was correlated with activity in the bilateral anterior insula cortices (AI). Peak voxel: ([27, 24, 0], z = 3.49, [−24, 27, 6], z = 3.66), p < 0.05, FWE-SVC. Image is at y = 25; dark blue p < 0.005, light blue p < 0.001. (Ci) Activity in the ACC was correlated more strongly with the chosen learned risk than chosen described risk (showed a positive correlation with the (chosen learned risk – chosen described risk) contrast). Peak voxel: ([12, 33, 30], z = 3.49), p < 0.05, FWE-SVC. This shows that risk-sensitive activity in these regions was selective for learned-value options. Image is at x = 13; red p < 0.005, orange p < 0.001. (Cii) Activity in the left AI correlated more strongly with chosen described risk than chosen learned risk (showed a negative correlation with the (chosen learned risk – chosen described risk) contrast). Peak voxel: ([−30, 33, 6], z = 3.59), p < 0.05, FWE-SVC. This shows that risk-sensitive activity in these regions was selective for described-value options. Image is at y = 25; dark blue p < 0.005, light blue p < 0.001. (D) Mean parameter estimates for activation in the ACC (red) and left AI (blue) for the (chosen learned risk – chosen described risk) contrast. This illustrates that activity in ACC was correlated more strongly with the value of learned cues than described ones, whereas the left AI showed the opposite pattern. (This presentation is for illustrative purposes only; black bars indicate 90% confidence intervals.)

If learned- and described-value estimates generated during risky decision making have distinct neuronal substrates, then we might expect this to be reflected in distinct influences of learned and described risk (here defined as outcome variance [20, 21]; Supplemental Experimental Procedures). Indeed, this prediction is supported by our RT data, which show a significant choice-condition-by-risk interaction, with learned risk having a greater impact on hastening subjects' responses (p < 0.001; Figure 2E; Table S1B). By examining ROIs previously associated with outcome risk and uncertainty [20–26], we again show differential patterns of activity. Risk-related activity reflecting choice of learned options (LR) was seen in the anterior cingulate cortex (ACC) in precisely the same region as that observed in previous studies involving learned uncertainty about the decision environment [22, 24, 25] (p < 0.05, family-wise error, small-volume corrected [FWE-SVC]; Figure 4A; Table S3). In contrast, the risk of selected described-value cues (DR) was correlated with activity in bilateral anterior insula cortices (AI) in regions previously reported as expressing risk in a task involving explicit assessment [21] (both p < 0.05, FWE-SVC; Figure 4B; Table S3). Analyzing the (LR-DR) and (DR-LR) contrasts indicated that these activation patterns differed significantly from one another in ACC and the left AI (both p < 0.05, FWE-SVC; Figures 4C and 4D; Table S3). At offer time, only temporally decaying risk-correlated activity was found (see Supplemental Data).

By testing for relative risk encoding using a post hoc ROI analysis similar to that described above, we found that activity in ACC showed a negative correlation with DR but was not statistically significant (p = 0.090 Bonferroni), whereas activity in the AI did not show a negative correlation with LR (Figure S3C). Our data are thus consistent with relative risk encoding in the ACC, but at the same time they do not provide strong support for this suggestion.

Both RT and imaging correlates of risk could, in principle, be explained by nonlinear value encoding rather than risk encoding per se. This is highly unlikely in the case of our imaging findings, because there is no overlap between brain regions correlated with value and risk; given the fit between our RT data and imaging, we suggest that this is not the most probable explanation here, either.

## Discussion

Neuroscientific studies of human decision making tend to situate themselves conceptually within one of two frameworks: learning theory (most commonly reinforcement learning [1, 19]) and behavioral economics (most often in the shape of prospect theory [12, 27–29]). Although it is conceivable that value estimates, based on different kinds of information, are treated equivalently at the neural level, here we show this is not the case. Instead, our data show that during decision making under risk, value estimates based on learned and described information evoke differential patterns of activity within value-sensitive regions. These results speak against the application of a single unifying theoretical framework to relate empirical findings concerning learning to those based on microeconomics.

The finding that activity in the vmPFC/OFC shows a strong positive response to learned value fits neatly with a large body of evidence linking this region with subjective valuation [11, 30–35], in particular the finding that the vmPFC/OFC encodes the value of a variety of different goods [30, 32, 35, 36], which is likely to depend upon prior experience of identical or similar goods. It also tallies with a more specific proposal derived from reinforcer devaluation studies, which indicate that the OFC is essential for using and updating outcome value [37–40].

It is less clear, by contrast, how precisely to interpret positive striatal responses to described value, because little prior work speaks directly to the issue of valuation by description. One possibility is that explicitly presented information has access to dopaminergic circuits akin to those involved in generating reward prediction errors [15, 41]. This is somewhat in tension with the finding that RT was related to LV but not DV, but there remains uncertainty about exactly what aspect of performance is mechanistically related to reaction time, which can be taken as a measure of both Pavlovian and instrumental responding.

In PCC, VP, and thalamus at choice time and in various regions at offer time, we find evidence of relative value encoding. Our data are consistent with this being the case also for vmPFC/OFC. This suggests that, rather than a strict anatomical dissociation, LV and DV processing may be reflected in differential sensitivities to these types of information in valuation regions. This can explain why prior studies, none of which force an explicit dissociation between LV and DV, report value-correlated activity across these regions (e.g., [12, 13]), because in these instances activity need reflect only a single value, irrespective of what type of information is used to generate it.

A similar point can be made in relation to our finding of differential sensitivity to learned and described risk in two areas previously implicated in encoding risk [20–26]. Existing literature indicates ACC risk-correlated activity in the context of learning [22, 24, 25] and insula activity where there is an explicit assessment of probabilities [20, 21, 23] (though feedback is often present in these latter experimental paradigms). However, at least one study has reported risk-related activity in both areas [26]. The activity patterns we observe here could again point to differential sensitivity to different kinds of risk information in a network of risk-sensitive areas rather than to an absolute anatomical dissociation.

A potential concern in our study is the fact that learned and described cues are not exactly matched, because there were more described than learned cues (nine compared with three) and because described cues were more novel than learned ones. We do not think either difference explains our results. On the one hand, it is unlikely that a jump from three to nine types of cue would radically alter valuation mechanisms, and in any case subjects effectively had to order a combined set of 12 cues rather than simply generate preferences within separate sets of three (learned) and nine (described) options. On the other hand, novelty responses also seem unlikely to explain our data, because there is no reason to suppose that they would covary parametrically with value. Additionally, we do not find any resemblances between temporally decaying and stable activity across the conditions, which would be expected if simple prior experience (as opposed to value learning) could explain our data.

Studying how evaluations are processed based on different kinds of information is of direct practical importance for understanding choice behavior in a range of real-life scenarios (e.g., medical decision making, financial trading). On this basis, we suggest that our results represent a modest first step toward understanding decision making in such complex but quotidian situations.

## Supplemental Information

Document S1. Supplemental Experimental Procedures, Supplemental Data, Three Tables, and Four Figures
