# Supplementary material for: Differentiable Neural Substrates for Learned and Described Value and Risk
Source: Curr Biol. 2010 Oct 26;20(20):1823–9. doi: 10.1016/j.cub.2010.08.048 (PMC2977067; doi:10.1016/j.cub.2010.08.048)
Supplement: Document S1. Supplemental Experimental Procedures, Supplemental Data, Three Tables, and Four Figures [file mmc1.pdf]

Current Biology, Volume *20*

Supplemental Information

Differentiable Neural Substrates for Learned and Described Value and Risk

Thomas H.B. FitzGerald, Ben Seymour, Dominik R. Bach, and Raymond J. Dolan

Supplemental Experimental Procedures

Subjects

18 (9 female) subjects, age range 19–27, all right handed, participated in the study. All subjects were free of neurological or psychiatric disease and fully consented to participate. The study was approved by the Joint National Hospital for Neurology and Neurosurgery (University College London Hospitals NHS trust) and Institute of Neurology (University College London) Ethics Committee. After scanning subjects received a sum of money according to their performance during the task.

Training Phase

Prior to performing the task, subjects underwent a training phase to ensure that they had adequately learnt the value of the three learnt value cues (Fig. S1). These cues consisted of a square, a triangle and a cross, each in a different colour (red, green, blue). This was fully counterbalanced across subjects. On any given trial of the training phase (except those discussed below) subjects were presented with two of the shapes, one on the left and one on the right of the screen. They selected one of these with a key press. The non-selected cue then disappeared, and subjects were presented with appropriate feedback: 'Win 10p' with probability p (where p=0.1, 0.5, 0.9), and 'Win 1p' with probability 1-p. (These two outcomes will henceforth be referred to as 'win' and 'no win' outcomes.) These probabilities were fixed throughout the experiment, something which was made clear to the subjects. Feedback was pseudorandomised, and each subject received precisely the same proportions of outcomes, though in a shuffled order. To ensure that subjects received the same amount of feedback about each cue, the training phase was divided into 60 trial sub-sessions, and once subjects had selected a particular cue 20 times it stopped appearing until all three cues had been selected 20 times. This meant that subjects sometimes made forced-choice decisions, where only one shape appeared. Note that in these cases they still had to pay attention to the cue, in order to select the correct button response. In order to minimise the generation of explicit rules during this phase (for example win counting) subjects performed a simple memory test. At the beginning of each sub-session they were shown three everyday words. They were asked to report these at the end of the block.

Subjects were informed that they would receive what they won in this phase of the experiment, but that the principle aim of this phase was to learn how good each of the three shapes was. They

were also told that once they had selected a particular cue a certain number of times it would disappear.

Subjects performed a long training phase consisting of four sub-sessions (240 trials in total) on a desktop PC outside the scanner, and then a shorter two-block session (120 trials) inside the scanner, during acquisition of fieldmaps and localisers. Out of scanner responses were indicated on the PC keyboard. In-scanner visual cues were presented on a computer monitor projected onto a screen, visible via an angled mirror on top of the fMRI head coil, and subjects indicated their choices by pressing one of two buttons on an MRI-compatible button box. To control for memory effects which might have been induced by seeing feedback about a particular cue more recently than others, subjects also performed a second two-block (120 trial) session just prior to task commencement where each trial involved a forced choice (only one cue was presented at a time). Thus subjects received a total of 160 trials of feedback per cue.

Task Phase

Subjects then proceeded to the main task. Here they were presented with a choice between two kinds of cue: one of three learnt value cues (LVCs), and one of nine described value cues (DVCs). These consisted in a pie chart with a number beneath, both indicating the precise probability of winning on that particular trial. (Win probabilities were 0.05, 0.1, 0.2, 0.4, 0.5, 0.6, 0.8, 0.9, 0.95) We avoided having the same number of LVCs and DVCs, as this would be likely to suggest to the subject an explicit figure for the LVCs. These were presented sequentially, each for 2000 ms, with a 500 ms gap (Fig.1), followed by a choice screen. Successful choices (those made during the 2000ms presentation of the choice screen) were indicated by the appearance of a circle around the selected option. Inter-trial intervals were jittered between 1000ms and 4000ms. Importantly, in this phase no outcome feedback was presented to subjects. This meant that no learning about the values of the explicit cues could take place. Subjects underwent a brief training phase (six trials) in which each of the learnt cues appeared twice, offered against explicit probabilities 0.25 and 0.75 (note that these described probabilities did not appear in the main task). This was then followed by two blocks of 108 trials in pseudorandomised order, in which each possible combination (n=27) occurred four times. The results of subjects' choices were recorded by the computer, and outcomes calculated according to the selected probabilities. In one subject (subject 14), equipment malfunction meant that data was recorded only from the first session. This was included within the group results. At the end of the experiment subjects received what they won in this second phase, together with a fixed amount for the learning phase.

This between-domain paradigm, mandating subjects choose between learnt and described value cues, was selected instead of previously used within-domain paradigms [4-8] in order to force a dissociation between value estimates in brain structures which might preferentially encode one or other signal, that under normal conditions act in concert and thus in a within-domain, or mixed paradigm, appear to be involved similarly in responding to, or generating, estimates of learnt and described value.

Post Task

After completion of the task, subjects were asked to fill in a short questionnaire in which they estimated the win percentage of each of the learnt value cues they had been presented with, and gave a certainty rating (from 1: Very uncertain to 10: Very certain) for each of these (Table S1A).

Behavioural Analysis

Subjects' choice patterns for each of the three LVCs were fitted separately with a logistic function using maximum likelihood estimation [36]. This allowed us to estimate the indifference points for each LVC in terms of described value, by taking the point at which the choice probabilities for both options were equal, allowing their direct comparison along a behaviourally derived scale. Risk was defined as outcome variance, and calculated for each cue as follows:

$$Risk = [(1 - P_W) * (M_N - EV)^2 + P_W * (M_W - EV)^2]$$

where P_W is the probability of winning, EV is the expected value of the gamble and M_W and M_N represent the magnitudes of outcome associated with winning and not winning (10p and 1p respectively). The results of these analyses were then used to construct parametric regressors for use in the fMRI analysis as described below. All behavioural analysis was performed using Matlab 7.1 (Mathworks, Natick MA).

After discarding error trials, the log-transformed reaction-time data from single subjects were fitted with a normal distribution, and then normalised by the single subject mean and standard deviation. Data were then pooled across subjects and subjected to a multiple regression analysis. The factors in the regression were which session (first or second) the trial was taken from, the absolute difference in subjective value between the options available on that trial (ΔV), whether subjects chose the described value cue or not (CD), the subjective value of the chosen option (CV), the interaction CV*CD, the risk of the chosen option (CR), and the interaction CR*CD, recursively orthogonalised in that order.

To generate Figure 2D we performed a multiple regression analysis incorporating only the first three parameters included in the main model (Session/ ΔV/CD). Analysing the residuals of this model allowed us to plot the effects of CV and CV*CD on reaction time with the effects of these three parameters removed. We then separated the trials, first according to which option-type subjects selected, and second according to whether the subjective value of that chosen option was low ($P \leq 0.33$), medium ($0.33 < P < 0.66$) or high ($P \geq 0.66$), producing a total of six bins. The RT data in each bin was then fitted with a Gaussian distribution using the Matlab normfit function, and results of this used to generate the mean values and confidence intervals shown. Figure 2E was created in an identical fashion, except that a model including the first five parameters of the original RT model (Session/ ΔV/CD/CV/CV*CD) was used, and the data was divided into two bins (low and high risk) in each condition.

fMRI

Gradient-echo T2*-weighted echo-planar (EPI) images were acquired on a 3T Trio Siemens AG (Erlangen, Germany) scanner. Scanner settings (TE: 0.07ms, TR 2.8s, 40 2mm slices acquired in descending order at an angle of 30° in the anterior-posterior axis) were designed to optimize

sensitivity in the OFC [S1]. 380 images were collected in each session (~18 mins each, two per subject). In one subject (Subject 14) equipment failure meant that data from only one session was recorded. Whole brain 1mm x 1mm x 1mm T1-weighted structural images were also acquired, coregistered with mean EPI images and averaged across subjects to permit anatomical localization. Subjects lay in the scanner with foam head-restraint pads to minimize any movement. They responded using an fMRI compatible button box.

Preprocessing and statistical analysis were carried out using SPM5 (Wellcome Trust Centre for Neuroimaging, London, www.fil.ion.ucl.ac.uk/spm). After discarding the first five images to allow for T1 equilibration effects, images were realigned with the first volume and unwarped using fieldmaps generated using the Fieldmap toolbox as implemented in SPM5 [S2,S3]. This corrects for both static distortions and motion-related alterations in these distortions. They were then coregistered with the individual subject's structural scans. Structural scans were segmented into grey and white matter and normalised to a standard template. This transformation was then applied to the coregistered EPI images, which were then smoothed using a 6mm full-width at half-maximum Gaussian kernel. Realignment parameters were inspected visually to identify any potential subjects with excessive head movement, and those with excessive movement (n=1) were removed from imaging analysis.

Primary Analyses

The images were analyzed in an event-related manner using the general linear model, with trials represented by a delta function. In the 'offer time' model, events were located at the time of presentation of the first option to the subject. (We only analysed the first offer to avoid any interaction between different the first and second offer on any given trial.) These events were modulated by 4 parametric regressors, one representing the learnt subjective value of the first cue on trials where the learnt cue was presented first (LV), together with its counterparts for described value (DV) and learnt and described risk (LR and DR). The second cue and choice screen onsets were entered as regressors of no interest. These regressors were orthogonalised in the order LV, DV, LR, DR using the Gram-Schmidt process.

In the 'choice time' model reported in the main text, events were timed at choice screen onset. These events were then modulated by 5 parametric regressors, representing the subjective values of the learnt and described cues presented on each trial, and the learnt and described risk of the option which subjects selected on a particular trial as well as the subject's reaction time (RT) for that trial, to control for the effects of movement and deliberation. We modelled the risk in this way because preliminary analysis suggested that it was a much better fit with risk-related activity in regions previously shown to encode statistical uncertainty [20,22]. Note that we do not make a strong claim about whether the differential activity patterns we observed were related to LR and DR representations which were present on each trial, or ones which were only generated if a subject chose that type of cue, and the distinction is not important for our primary conclusions. Cue onsets were entered as regressors of no interest. These regressors were orthogonalised in the order RT, LV, DV, LR, DR using the Gram-Schmidt process.

The resulting stimulus functions were then convolved with a haemodynamic response function (HRF). Regression was performed using restricted maximum likelihood estimations in SPM.

Effects of no interest included error trials. Low-frequency fluctuations were removed using a high-pass filter (cut-off 128s) and an AR(1) model plus white noise was used to correct for temporal autocorrelation. Group-level activation was determined by performing a one-sample *t* test on the linear contrasts of the statistical parametric maps (SPMs) generated during the first level analysis.

Analysis of Temporally Modulated Activity

To model effects of temporal decay on value representations we first created a temporal modulator of the form

$$M_t = \exp(-t/\tau)$$

where t corresponds to the trial number and τ is a constant controlling the rate of decay. We then created temporally modulated value and risk parameters for each model by first detrending and multiplying the relevant parameter and the temporal modulator. Additional analysis models were then created which were identical to those described above, except that they included the relevant temporal and temporal*parametric modulators. In each case, the regressors were orthogonalised in the order temporal modulator, parametric modulator, temporal*parametric modulator.

We modelled the data using $\tau = 20$ and $\tau = 40$, which were selected to produce significant and rapid decay in the strength of parametric modulation. Both produced extremely similar results, and we report results with $\tau = 40$.

Supplementary Choice Time Analysis

We hypothesised that LV and DV would be compared in the vmPFC/OFC and PCC [36]. In order to test this we generated a model similar to the choice time model described above, but containing only two parametric regressors, RT and, orthogonalised to it, the absolute difference in subjective value between the cues presented on that trial (ΔV). We applied the same significance criteria as in the main model. Because LV was also correlated with activity in the vmPFC/mOFC we calculated a check model in which LV was orthogonalised to ΔV, and observed the same pattern of LV activations (ΔV results from this latter model are reported in the Supplemental Data and in Table S2).

Check Models

To ensure our results were not confounded by other factors, and to explore additional valuation parameters which we hypothesised might be correlated with brain activity, we analysed the data using a number of extra check models. These included the subjective uncertainty associated with each learnt cue value as indexed by (a) the softmax temperature parameter associated with the behavioural results for each learnt cue for each individual subject, (b) the certainty level reported by subjects on the post-test questionnaire, and (c) the degree to which they deviated from optimal in their estimated subjective valuations. We found no significant uncertainty-related results in our dataset.

Additionally we tested models including the subjective value of the chosen option on each trial. This was correlated with activity in the vmPFC/OFC and nucleus accumbens, but, since chosen value and ΔV are strongly correlated in this task, we checked these results in a model in which both chosen value and ΔV were present, without orthogonalisation [36]. This picks out only those regions which show activity significantly correlated with independent components of the two parameters. In these models, ΔV correlated activity was still present in the vmPFC/OFC, but no clusters survived multiple comparisons correction in the chosen value contrast, and these results are thus not discussed further.

Statistical Thresholding

We report results which were significant at $P < 0.05$ whole brain cluster-corrected, with individual voxels thresholded at $Z > 2.3$. Additionally we specified contrast-specific Regions of Interest (ROIs) based on previous findings as detailed below. We report results in these regions which were significant at $P < 0.05$ cluster-corrected using a small volume correction (SVC) for the ROI.

Preliminary analyses of the principal risk-related activations suggested more focal patterns of activity, which were not well-captured by testing for cluster-size. Thus to analyse these contrasts we instead used the complementary method of examining peak activations. Here our criterion for significance was set as $P < 0.05$ family wise error corrected (FWE), again applying a small-volume correction for our *a priori* ROIs. Although no confound is introduced by this process, we acknowledge that the need to deviate from our primary mode of analysis should be taken as a *caveat* concerning these results.

ROIs were specified as 10mm spheres centred on activation peaks reported by previous studies. They were set in the vmPFC/OFC (MNI coordinates: [-3, 45, -18], Study: [11]), PCC ([-3, -46, 38], [12]), midbrain ([0, -21, -30], [S4]) and ventral striatum ([-28, 8, -6] / [28, 8, -6], [15]) for the value contrasts, and in ACC ([8, 26, 36] / [-8, 26, 36], [24]) and AI ([35, 26,4] / [-35, 26, 4], [20]) for the risk contrasts.

Activation coordinates are given in Montreal Neurological Institute (MNI) space as generated by SPM5. Images displaying results examined with cluster-correction are thresholded at $P < 0.05$ whole-brain/small-volume corrected. Other images are thresholded at $P < 0.005$ uncorrected to illustrate the extent of activation.

Post-Hoc Analyses

To explore whether subjects' overvaluing of low learnt probability cues was related to activity in the regions identified as displaying a sensitivity to value at choice time, we assessed the correlation between single subject parameter estimated for the (LV-DV) contrast and individual subjects' valuations of low learnt probability cues in three regions of interest (vmPFC/OFC, PCC, left VP) determined by our main results. ROIs were derived from significantly (LV-DV) clusters (vmPFC/OFC, PCC) and (DV-LV) clusters (left VP) generated by our main analysis. (Note this constitutes an independent selection of voxels for the correlation analysis) Single subjects' parameter estimates for these regions were then derived using the MarsBaR SPM toolbox:

(http://marsbar.sourceforge.net/). The significance of the observed correlation was assessed using a permutation test in which it was compared with a surrogate distribution of correlations generated by shuffling the low probability learnt cue estimates between subjects 2000 times. Because this procedure generates only a single value for each ROI a Bonferroni correction for only three comparisons was needed. To generate the best fit line shown in Fig.3E a linear regression was performed using the *regstats* function in Matlab. To ascertain whether the correlation between overvaluing and single-subject (LV-DV) parameters was driven by increased positive responses to LV, increased negative responses to DV, or both, we calculated the correlation in the vmPFC/OFC ROI between individual LV and DV parameter estimates and behavioural overvaluing, assessing significance using an identical permutation test. Note that this is a *post hoc* test, and is thus illustrative rather than definitive.

To generate Figure 3D we used MarsBar to extract single-subject parameter estimates for the (LV – DV) contrast in the vmPFC/OFC and left VP. These regions were defined by the significantly activated clusters in these regions found in the primary analysis. To generate Figure 4D we used MarsBar to extract single-subject parameter estimates for the (LR – DR) contrast in the ACC and left AI. These regions were defined as by the clusters of activated voxels in these regions found in the primary analysis at $P < 0.005$ uncorrected. To generate Figure S4 we generated an additional model in which trials were divided up into three bins on an individual subject level according to whether the difference in estimated subjective value between the options available was in the range $p \leq 0.33$, $0.33 < p < 0.66$, or $p \geq = 0.66$. MarsBar was then used to extract single-subject mean parameter estimates for the significantly activated vmPFC/OFC cluster found in supplementary model 1. Note that the purpose of these figures is illustrative only, and they have not been used as the basis for any statistical inference.

Relative Value Encoding

To assess whether activity patterns at choice time were best explained by a strict anatomical dissociation between regions processing different kinds of reward information, or by relative value encoding in discrete brain regions, we performed a qualitative *post hoc* ROI analysis to index whether activity in the vmPFC/OFC and PCC was negatively correlated with DV, and if activity in the VP and thalamus was negatively correlated with LV. We used ROIs taken from the functionally activated clusters (threshold $P < 0.01$ uncorrected) for the (LV-DV) contrast in the vmPFC/OFC and PCC and for the (DV-LV) contrast in the VP and thalamus. We performed a similar analysis for value at offer time, taking ROIs from the (DV-LV) contrast in the ACC, PCC and cerebellum, and for risk at choice time, taking ROIs in the AI and ACC.

Note that in the case of offer time analysis, because we only model the first offer and never capture LV and DV on the same trial, we can only infer relative value encoding from regions which showed one pattern of responding to learnt-value cues when they were presented first, and the opposite to described-value cues when they were presented first.

Supplemental Data

Cue Valuations

All subjects correctly ordered the values of the LVCs (see Table S1A). Averaging all the separate parameter estimates suggested that subjects tended to overestimate the probability of the smallest LVC (μ = 0.23, P < 0.005 two-tailed t-test), but that their estimates were not significantly different from the actual probability for the two larger LVCs (μ = 0.54, P = 0.13, and μ = 0.90, P = 0.93 respectively). These tendencies were visible in the pooled choice data from the entire study, which yielded estimates of 0.23, 0.54 and 0.91 respectively (see Figure 1A).

A similar pattern was seen for the questionnaire results, and significant between-subject correlations were observed when comparing self-reported valuations and the estimates generated by the logit analysis. (Low LVC: R = 0.907, P < 0.001, Middle LVC: R = 0.759, P < 0.001, High LVC: R = 0.815, P < 0.001, All LVCs: R = 0.922, P < 0.001). (Significance was assessed using a permutation test in which the observed data were shuffled 1000 times and the recalculated correlations used to generate a surrogate distribution)

Neural Correlates of Low LVC Overvaluing

Single subject parameter estimates for the Learnt Value – Described Value contrast in vmPFC/OFC showed a significant positive correlation with subjects' valuation of low probability learnt cues (R = 0.644, P = 0.006, Bonferroni corrected). A correlation was seen in PCC which did not survive multiple comparison correction (R = 0.430, P = 0.119, Bonferroni corrected), whilst no significant evidence of a correlation was seen in the left VP (R = 0.153, P = 0.810, Bonferroni corrected).

Offer Time Analyses

At offer time there were no regions where learnt value was correlated with brain activity at a conventional level of significance. However, lowering our threshold revealed two bilaterally symmetrical regions of activity at the anterior border of the caudate nucleus (both P<0.001 uncorrected, peak voxel analysis) (Table S2). We report these data descriptively because of their symmetry and location to regions strongly linked to value learning. (This activation showed no overlap with the VP activity correlated with described value at choice time) By contrast, described value correlated with activity in regions of midbrain overlapping the substantia nigra and ventral tegmental area (P < 0.05 ROI cluster-corrected), the PCC, ACC, right hippocampus, regions of the cerebellum and occipital cortex (all P < 0.05 whole-brain cluster corrected) (Fig. S2B). Of these findings, activity in ACC, PCC and cerebellum showed a significantly greater correlation with DV than LV, as assessed by the (DV–LV) contrast (Fig. S2C), suggesting that the neuronal value mechanisms at this stage differed. (Note that (DV-LV) and (LV-DV) responses cannot directly represent relative value signals, since we analyse activity at the presentation of the first cue alone, when only one kind of value information is available to the subject. Thus, evoked responses seen here are used solely to establish an overall profile of value related activity) Several regions were more active when subjects were presented with described

rather than learnt value cues (Table S2) though we urge caution as the cues differ systematically along a number of dimensions other than value (for example, appearance and familiarity).

Several regions showed activity consistent with relative value encoding in that significant negative correlations with LV were found in all three regions (ACC: $P = 0.016$, PCC: $P = 0.004$, Cerebellum: $P = 0.004$) (Fig. S3A). This suggests that activity in these regions reflects both LV and DV, but treated in different ways, consistent with the idea of differential sensitivities to value based on access to different kinds of information by valuation sensitive brain regions .

Temporally Decaying Activity

At offer time, temporally decaying learnt value signals were found in two clusters in the left superior temporal gyrus extending into the posterior insula and postcentral gyrus (both P<0.05 whole-brain cluster corrected). No regions displayed a temporally decaying value signal that survived multiple comparisons, but a region of the right insula showed a strong trend towards significance ($P = 0.09$, whole-brain cluster-corrected). No regions showed decaying value-correlated activity at choice time.

At offer time, no activity showed a significant correlation with risk, except for clusters in the left postcentral and right middle temporal gyri which showed decaying activity correlated with described risk ($P < 0.05$ whole-brain cluster corrected) (Table S2). These were not significantly different between conditions. At choice time, activity in one cluster including the left superior temporal gyrus and posterior insula and another in the right thalamus showed a rapidly decaying response to learnt risk ($P < 0.05$ whole-brain cluster corrected) (Table S2). There was no overlap at a threshold of $P < 0.01$ uncorrected between this activity in the posterior insula and the AI activity which was correlated with described risk.

Comparative Value (ΔV)

Previous work has suggested the existence of a signal in the vmPFC/OFC reflecting the comparative value of (the unsigned magnitude of the difference in subjective value between) dissimilar objects (ΔV) [36, S5]. We extend this finding here, showing that both RTs and activity in the vmPFC/OFC are strongly correlated with the comparative subjective value of learnt and described cues ($P < 0.001$ whole-brain cluster corrected) (Fig. S4, Tables S1B,2). This vmPFC/OFC activity is distinct from that correlated with LV (see Methods), and supports a role for this area in choosing between options which can only be evaluated based on different kinds of value information. It may thus represent a ‘final common valuation pathway’ in decision-making [S6].

Table S1A. Estimated Subjective Values and Questionnaire Results, Related to Figure 2

	**Learnt Value Cue 1**			**Learnt Value Cue 2**			**Learnt Value Cue 3**		
**Subject**	**S.V.**	**Q.W.**	**Q.C.**	**S.V.**	**Q.W.**	**Q.C.**	**S.V.**	**Q.W.**	**Q.C.**
**1**	0.16	12.5	8	0.70	65	6	0.85	85	8
**2**	0.37	40	8	0.45	60	8	0.95	99	9
**3**	0.36	40	6	0.52	60	7	0.74	75	7
**4**	0.42	41	6	0.50	44	6	0.94	91	8
**5**	0.35	40	5	0.72	70	6	0.74	85	5
**6**	0.20	10	3	0.50	45	3	0.79	90	7
**7**	0.01	5	6	0.57	55	7	1.00	95	9
**8**	0.08	30	4	0.43	50	6	1.00	95	8
**9**	0.12	10	7	0.27	30	6	1.00	95	9
**10**	0.30	40	6	0.53	60	6	0.93	85	8
**11**	0.11	15	7	0.55	60	7	0.90	90	9
**12**	0.54	50	(7*)	0.56	60	(7*)	1.00	95	(7*)
**13**	0.49	60	5	0.59	75	6	1.00	95	8
**14**	0.21	20	(6*)	0.60	55	(6*)	0.93	90	(6*)
**15**	0.05	1	8	0.50	45	8	0.86	90	9
**16**	0.20	15	8	0.56	60	5	0.82	85	9
**17**	0.11	0	9	0.57	50	9	0.90	90	9
**18**	0.00	3	6	0.56	65	5	0.81	85	8
Mean	***0.23***	***24***	***6.4***	***0.54***	***56***	***6.3***	***0.90***	***90***	***8.1***
Median	***0.20***	***17.5***	***6.4***	***0.56***	***60***	***6.3***	***0.92***	***90***	***8.1***

Left-hand column for each cue (S.V.): Estimated subjective value for each of the three learnt value cues from single subject logit analyses. The lowest value cue is significantly overvalued (P = 0.0047 two-tailed one sample t-test)

Middle and right-hand column for each cue: Post-test questionnaire results showing subjects' estimates of the win percentage of each cue (Q.W.), and the subjective certainty they gave to this estimate (Q.C.). Questionnaire estimates show a strong correspondence to the subjective value estimates given by the logit analysis. In particular they display a similar marked overvaluing of the lowest value learnt value cue (P = 0.0054 two-tailed one sample t-test). (*Indicates subjects who entered only one certainty score into the questionnaire. These scores are not included in the mean values given at the bottom, and these subjects were excluded from imaging analysis in the check models described in the Methods).

Table S1B. RT Analysis, Related to Figure 2

Parameter	Parameter Estimate	*P* value
Constant Term	0.3729	0.0033*
Session	-0.2435	0.0016*
Difference in Subjective Value (ΔV)	-0.5131	0.0000*
Chosen Described Cue (CD)	0.0172	0.6430
Chosen Value (CV)	-0.1306	0.1564
(CV)(CD)*	-0.4160	0.0057*
Chosen Risk (CR)	-0.0010	0.7246
(CR)(CD)*	-0.0405	0.0001*

Multiple regression analysis of normalised log-RTs. Significant reaction time decreases were correlated with session order, the difference in subjective value, and the interactions of the value and risk of the chosen cue with the type of cue which had been selected. This suggests that described value and described risk both had a stronger (facilitating) effect on behavioural responding than learnt value and risk. (* indicates significant effect at $P < 0.01$.)

Table S2. Significantly Activated Clusters, Related to Figure 3

Region	*Cluster Size*	*Side*	*Cluster Peak MNI [X Y Z]*	*Cluster Peak Z*
Learnt Cue Presentation – Described Cue Presentation (Offer Time)				
Inferior Temporal Gyrus	206	Right	51 -57 -24	4.19
Motor Cortex	264	Left	-48 -3 51	4.16
Caudate Nucleus	210	Bilateral	6 6 15	4.13
Middle / Inferior Temporal Gyri	173	Left	-36 -57 -3	3.71
Lateral Orbitofrontal Cortex / Anterior Insula	197	Left	-33 36 0	3.69
Learnt Value (Offer Time)				
Caudate Nucleus[+]	9	Left	-15 27 3	3.54
Caudate Nucleus[+]	12	Right	15 27 3	3.38
Described Value (Offer Time)				
Occipital Cortex / Cerebellum	688	Bilateral	27 -87 -12	3.93
Posterior Cingulate Cortex	235	Bilateral	15 -54 33	3.70
Hippocampus	217	Right	18 -33 0	3.68
Midbrain*	94	Bilateral	-3 -21 -18	3.66
Cerebellum	173	Left	30 -66 -21	3.47
Anterior Cingulate Cortex	155	Bilateral	15 48 15	3.27
Learnt Value – Described Value (Offer Time)				
-	-	-	-	-
Described Value – Learnt Value (Offer Time)				
Anterior Cingulate Cortex	308	Bilateral	9 30 36	4.03
Cerebellum	110	Right	12 -57 30	3.95
Posterior Cingulate Cortex	203	Bilateral	6 -66 27	3.54
Motor Cortex	108	Left	-51 -12 33	3.36
Learnt Value*Temporal Modulator (Offer Time)				
Superior Temporal Gyrus	117	Left	-42 -30 0	3.32
Postcentral Gyrus	156	Left	-54 -6 33	3.10
Described Value*Temporal Modulator (Offer Time)				
Insula[+]	84	Right	39 18 -6	3.66
(Learnt Value – Described Value)*Temporal Modulator (Offer Time)				
Lingual Gyrus	182	Bilateral	3 -69 0	3.74
Superior Temporal Gyrus / Postcentral Gyrus	178	Left	-54 -6 24	3.66
(Described Value – Learnt Value)*Temporal Modulator (Offer Time)				
-	-	-	-	-

Learnt Value (Choice Time)				
Medial Orbitofrontal Cortex	203	Bilateral	-15 57 -3	4.20
Posterior Cingulate Cortex	29	Bilateral	-3 -48 33	2.62
Described Value (Choice Time)				
Putamen	217	Left	-30 12 -6	4.29
Cerebellum	177	Bilateral	0 -63 -48	4.00
Putamen	177	Right	27 0 6	3.60
Learnt Value – Described Value (Choice Time)				
Posterior Cingulate Cortex	119	Bilateral	-9 -48 30	3.22
Medial Orbitofrontal Cortex*	53	Bilateral	-15 57 -3	3.66
Described Value – Learnt Value (Choice Time)				
Thalamus	173	Bilateral	0 -36 6	4.07
Putamen	108	Left	-27 9 0	3.17
Learnt Value*Temporal Modulator (Choice Time)				
-	-	-	-	-
Described Value*Temporal Modulator (Choice Time)				
-	-	-	-	-
Difference in Value (Choice Time)				
Medial Orbitofrontal Cortex	532	Bilateral	3 33 -6	4.21
Difference in Value*Temporal Modulator (Choice Time)				
-	-	-	-	-
Learnt Risk*Temporal Modulator (Offer Time)				
-	-	-	-	-
Described Risk*Temporal Modulator (Offer Time)				
Postcentral Gyrus	119	Left	-36 -18 45	3.49
Middle Temporal Gyrus	158	Right	51 -69 12	3.27
(Learnt Risk - Described Risk)*Temporal Modulator (Offer Time)				
-	-	-	-	-
Chosen Learnt Risk*Temporal Modulator (Choice Time)				
Middle Temporal Gyrus / Posterior Insula	104	Left	-48 -18 -18	3.57
Thalamus	96	Right	15 -24 3	3.18
Chosen Described Risk*Temporal Modulator (Choice Time)				
-	-	-	-	-
(Chosen Learnt Risk - Chosen Described Risk)*Temporal Modulator (Choice Time)				
Postcentral Gyrus	130	Right	36 -30 51	3.90
Supplementary Motor Area	195	Right	18 -21 51	3.71

The significantly activated clusters according to the cluster-size analysis. Activations correspond to figures **3A,B,C, S2A,B,C, S4**. All clusters were significant at $P < 0.05$ whole brain corrected except those marked * which were significant at $P < 0.05$ cluster corrected within *a priori* ROIs, and those marked $^{+}$ which showed only a trend towards significance.

Table S3: Peak Analysis of Risk-Related Neuronal Activity, Related to Fig.4

A: Positive Correlation with Chosen Learnt Risk (Choice Time)					
Region	*BA*	*Side*	*Voxels*	*MNI [X Y Z]*	*Peak Z*
Thalamus	-	Right	10	12 -3 -3	3.96
**Insula**	**48**	**Left**	**25**	**-24 27 6**	**3.66**
**Insula**	**47**	**Right**	**17**	**27 24 0**	**3.49**
Middle Frontal Gyrus, Orbital Part	47	Right	14	33 57 -3	3.40
Anterior Cingulate Gyrus	32	Left	9	-12 24 21	3.40
Posterior Lobe	-	Right	4	39 -66 -51	3.29
White Matter	-	Left	4	-39 -15 27	3.22
Lingual Gyrus	18	Right	7	15 -90 -6	3.16
Brainstem	-	Midline	7	0 -33 -18	3.15
Posterior Lobe	-	Right	4	27 -78 -45	3.07
Insula	47	Right	5	39 30 6	2.97
Posterior Lobe	-	Left	4	-15 -81 -18	2.97
Frontopolar Cortex	10	Left	4	-27 60 12	2.95
Fusiform Gyrus	19	Left	8	-33 -78 -15	2.90
Calcarine Sulcus	18	Right	11	3 -78 15	2.82
Insula	48	Right	4	39 15 0	2.81
Midbrain	-	Right	5	9 -24 -9	2.73
B: Positive Correlation with Chosen Described Risk (Choice Time)					
Region	*BA*	*Side*	*Voxels*	*MNI [X Y Z]*	*Peak Z*
Supplementary Motor Area	6	Left	14	-9 -6 57	3.7
Inferior Temporal Gyrus	37	Right	8	54 -51 -6	3.52
**Anterior Cingulate Gyrus**	**32**	**Right**	**4**	**12 33 30**	**3.49**
Precentral Gyrus	6	Left	14	-33 -9 57	3.48
Middle Temporal Gyrus	37	Right	17	63 -57 6	3.44
Middle Temporal Gyrus	37	Right	5	57 -63 18	3.19
C: Positive Correlation with (Chosen Learnt Risk – Chosen Described Risk) (Choice Time)					
Region	*BA*	*Side*	*Voxels*	*MNI [X Y Z]*	*Peak Z*
Supplementary Motor Area	6	Left	14	-9 -6 57	3.7
Inferior Temporal Gyrus	37	Right	8	54 -51 -6	3.52
**Anterior Cingulate Gyrus**	**32**	**Right**	**4**	**12 33 30**	**3.49**

Precentral Gyrus	6	Left	14	-33 -9 57	3.48
Middle Temporal Gyrus	37	Right	17	63 -57 6	3.44
Middle Temporal Gyrus	37	Right	5	57 -63 18	3.19
D: Positive Correlation with (Chosen Described Risk – Chosen Learnt Risk) (Choice Time)					
Region	*BA*	*Side*	*Voxels*	*MNI [X Y Z]*	*Peak Z*
Thalamus	-	Right	12	12 -3 -3	4.91
**Insula**	**48**	**Left**	**9**	**-30 33 6**	**3.59**
Middle Frontal Gyrus, Orbital Part	11	Right	8	33 60 -3	3.45
Anterior Lobe	-	Right	14	12 -39 -18	3.24
White Matter	-	Right	9	3 21 12	3.19
Midbrain	-	Right	5	9 -27 -9	3.16
Brainstem	-	Left	6	-3 -33 -18	3.12
Insula	47	Right	5	30 27 3	2.95
Anterior Lobe	-	Left	4	-3 -51 -15	2.65

Text in bold indicates regions which reached statistical significance at $P<0.05$ FWE corrected in predefined regions of interest. * indicates $P<0.05$ whole-brain corrected elsewhere.

S3A: Regions correlated with Chosen Learnt Risk. The activations correspond to figure **4A** in the main text.

S3B: Regions correlated with Chosen Described Risk. The activations correspond to figure **4B** in the main text.

S3C: Regions correlated with (Chosen Learnt Risk – Chosen Described Risk). The activations correspond to figure **4Ci** in the main text.

S3D: Regions correlated with (Chosen Described Risk – Chosen Learnt Risk). The activations correspond to figure **4Cii** in the main text.

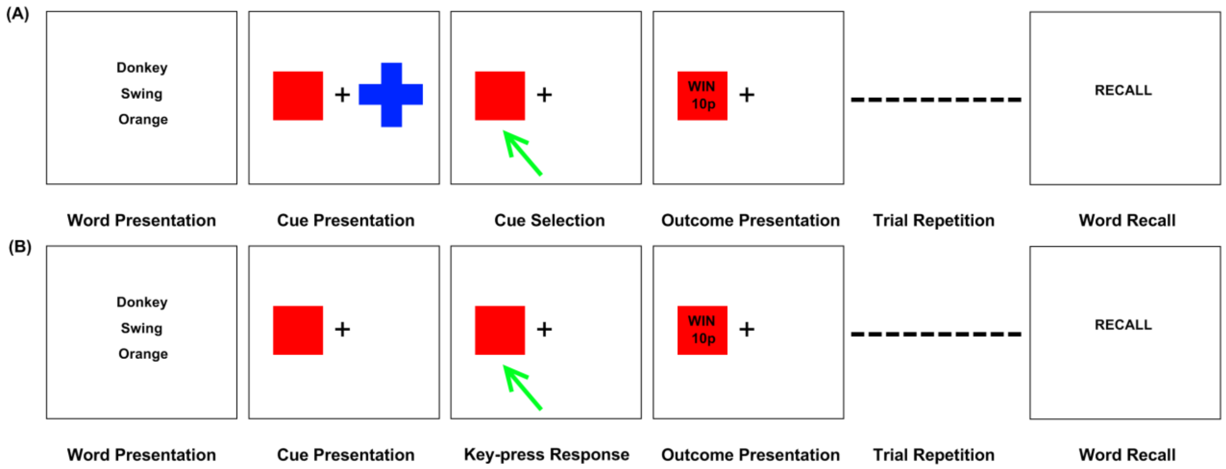

Figure S1. Training Paradigm, Related to Figure 1

Illustration of both kinds of block of the training paradigm. At the start of a block subjects were presented with three words to remember. In the first type of block, illustrated by (A) they were then presented with a series of trials in which two cues were presented and they had to select one. After selection the non-selected cue disappeared, and, after a 400 ms delay, the trial outcome was presented to the subject for 2000 ms. The second type of block illustrated by (B) was identical except that only one cue was presented at a time, and subjects merely had to make an instrumental response rather than a choice.

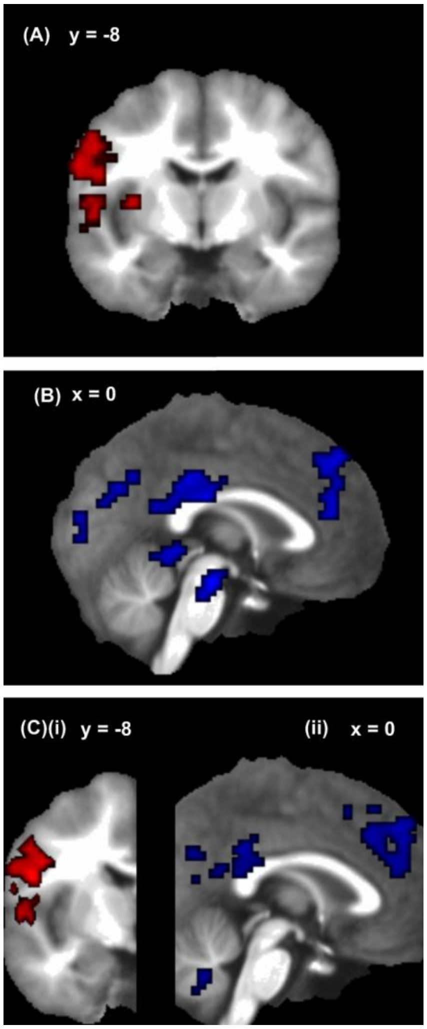

Figure S2. Activity at Offer Time, Related to Figure 3

(A) This shows that temporally decaying learnt value at offer time correlated with activity in the superior temporal gyrus, posterior insula and postcentral gyrus Superior temporal gyrus/posterior insula: peak cluster voxel ([-42 -30 0], z=3.32), (*P*<0.05 whole-brain cluster corrected). Postcentral gyrus: peak voxel ([-54 -6 33], z=3.10), (*P*<0.05 whole-brain cluster corrected) (Image is at y=-8)

(B) Described value at offer time correlated with activity in the midbrain, PCC, ACC, right hippocampus, regions of the cerebellum and occipital cortex. Midbrain: peak voxel ([-3 -21 -18], z=3.66) (*P*<0.05 cluster-corrected for the midbrain ROI). PCC: peak voxel ([15 -54 33], z=3.70), (*P*<0.05 whole-brain cluster corrected). ACC: peak voxel ([15 48 15], z=3.27), (*P*<0.05 whole-brain cluster corrected). Hippocampus: peak voxel ([18 -33 0], z=3.68), (*P*<0.05 whole-brain cluster corrected). Occipital cortex/cerebellum: peak voxel ([27 -87 -12], z=3.93) (*P*<0.05 whole-brain cluster corrected). Cerebellum: Hippocampus: peak voxel ([30 -66 -21], z=3.47), (*P*<0.05 whole-brain cluster corrected). (Image is at x=0)

(Ci) A direct comparison between responses to temporally modulated learnt and described value at offer time (the (Learnt Value*Temporal Modulator – Described Value*Temporal Modulator) contrast) shows that activity in the superior temporal and postcentral gyri was greater for temporally decaying learnt than temporally decaying described value. Superior temporal gyrus:

peak cluster voxel ([-42 -30 0] z=3.32), (*P*<0.05 whole-brain cluster corrected). Postcentral gyrus: peak cluster voxel ([-54 -6 33] z=3.10), (*P*<0.05 whole-brain cluster corrected). (Image is at y=-8)

(Cii) The (Described Value - Learnt Value) contrast at offer time shows that activity in ACC, PCC and cerebellum was better correlated with described than learnt value. PCC: peak voxel ([6 -66 27], z=3.54), (*P*<0.05 whole-brain cluster corrected). ACC: peak voxel ([9 30 36], z=4.03), (*P*<0.05 whole-brain cluster corrected). Cerebellum: peak voxel ([12 -57 30], z=3.95) (*P*<0.05 whole-brain cluster corrected). (Image is at x=0)

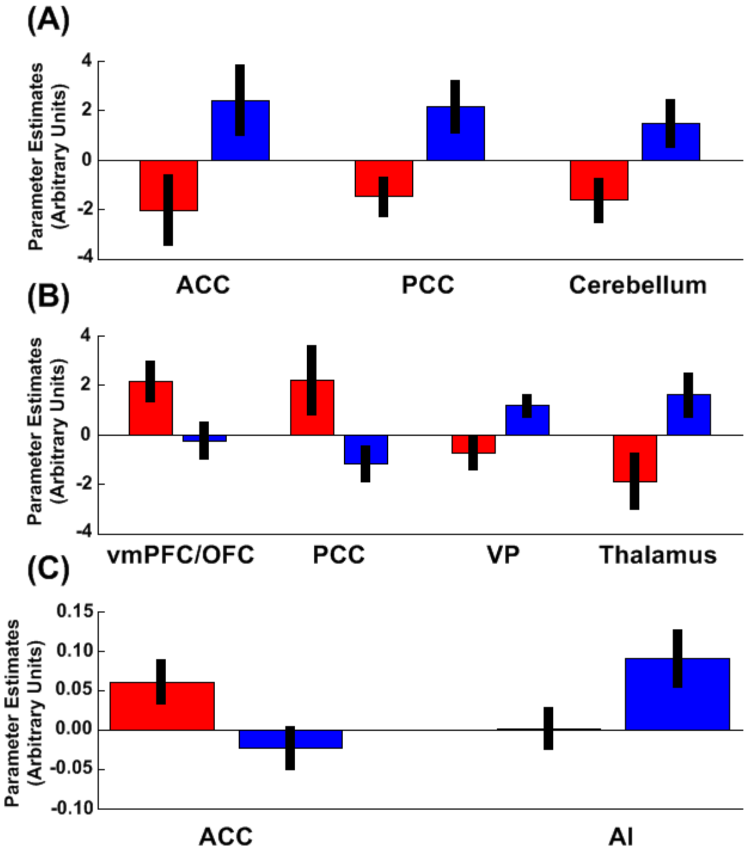

Figure S3. Parameter Estimates for the Relative Value Analysis, Related to Figures 3 and 4
Parameter estimates (arbitrary units) for LV/LR (red) and DV/DR (blue) in each ROI used in the relative value analysis. (This analysis is illustrative rather than conclusive, since we do not use unbiased ROIs. Black bars indicate 90% confidence intervals)

(A) ACC, PCC and cerebellum LV and DV parameter estimates at offer time.

(B) vmPFC/OFC, PCC, VP and thalamus LV and DV parameter estimates at choice time.

(C) ACC and AI LR and DR parameter estimates at choice time.

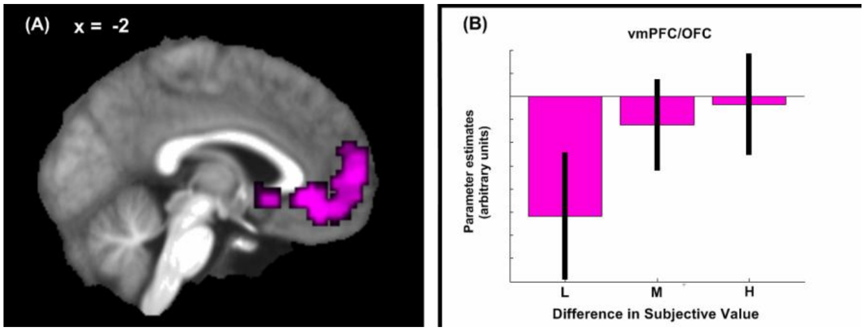

Figure S4. Comparative Value in the vmPFC/OFC, Related to Figures 3 and 4
(A) The difference in subjective value was significantly correlated with activity in a single cluster in the vmPFC/OFC. Peak voxel: ([3, 33, -6], z=4.21). (P<0.001 whole-brain cluster corrected) (Image is at x=-2)
(B) Mean parameter estimates in arbitrary units for the vmPFC/OFC cluster for low (0-0.33), medium (0.33-0.66) and high (0.66-1) difference in value trials, illustrating that activity in this region was significantly correlated with the difference in subjective value between the options presented to subjects on a particular trial. (This presentation is for illustrative purposes only, black bars indicate 90% confidence intervals)

Supplemental References

S1. Deichmann R., Gottfried J.A., Hutton C., and Turner R. (2003). Optimized EPI for fMRI studies of the orbitofrontal cortex**.** Neuroimage. *19***:** 430-441.
S2. Hutton C., Bork A., Josephs O., Deichmann R., Ashburner J., and Turner R. (2002). Image Distortion Correction in fMRI: A Quantitative Evaluation**.** Neuroimage *16***:** 217-240.
S3. Hutton C., Deichmann R., Turner R., and Andersson J.L.R. (2004). Combined correction for geometric distortion and its interaction with head motion in fMRI**.**.
S4. O'Doherty J.P., Buchanan T.W., Seymour B., and Dolan R.J. (2006). Predictive Neural Coding of Reward Preference Involves Dissociable Responses in Human Ventral Midbrain and Ventral Striatum**.** Neuron *49***:** 157-166.
S5. Boorman E.D., Behrens T.E.J., Woolrich M.W., and Rushworth M.F.S. (2009). How Green Is the Grass on the Other Side? Frontopolar Cortex and the Evidence in Favor of Alternative Courses of Action**.** Neuron *62***:** 733-743.
S6. Platt M.L. and Glimcher P.W. (1999). Neural correlates of decision variables in parietal cortex**.** Nature *400***:** 233-238.
